# Multiple chronic disorders - health care system’s modern challenge in the Maccabi Health Care System

**DOI:** 10.1186/2045-4015-3-29

**Published:** 2014-08-29

**Authors:** Jonathan E Arbelle, Gabriel Chodick, Alex Goldstein, Avi Porath

**Affiliations:** 1 Medical Division, Maccabi Healthcare Services, Tel Aviv, Israel; 2 Faculty of Medicine, Ben-Gurion University, Beer Sheva, Israel; 3 Faculty of Medicine, Tel-Aviv University, Tel Aviv, Israel

## Abstract

**Background:**

One of the major challenges health care systems face in modern time is treating chronic disorders. In recent years, the increasing occurrence of multiple chronic disorders (MCC) in single individuals has compounded the complexity of health care. In 2008, it was estimated that worldwide as many as one quarter of the population between the ages of sixty five to sixty nine suffered from two or more chronic conditions and this prevalence rose with age. Clinical guidelines provide guidance for management of single disorders, but not for MCC. The aim of the present study was the study of the prevalence, distribution and impact of MCC in a large Israeli health system.

**Methods:**

We performed a cross-sectional study of MCC in the Maccabi Healthcare System (MHS), Israel’s second largest healthcare service, providing care for approximately two million people. Data regarding chronic conditions was collected through electronic medical records and organizational records, as was demographic and socioeconomic data. Age and sex specific data were compared with previously published data from Scotland.

**Results:**

Two thirds of the population had two or more chronic disorders. This is significantly higher than previously published rates. A correlation between patient age and number of chronic disorders was found, as was a correlation between number of chronic disorders and low socioeconomic status, with the exception of children due to a high prevalence of learning disabilities, asthma, and visual disturbances.

**Discussion:**

MCC is very prevalent in the MHS population, increases with age, and except for children is more prevalent in lower socioeconomic classes, possibly due to the a combination of the structure of the Israeli universal insurance and requirements of the ministry of education for exemptions and benefits. A higher than previously reported prevalence of MCC may be due to the longtime use of use of integrated electronic medical records.

**Conclusions:**

To effectively deal with MCC health care systems must devise strategies, including but not limited to, information technologies that enable shared teamwork based on clinical guidelines which address the problem of multiple, as opposed to single chronic disorders in patients.

## Background

In recent years, ageing of the world population combined with the effects of urbanization and globalization, an increase in communicable and non-communicable disorders, patients presenting with multiple complex diseases has occurred [1]. To effectively deal with chronic disorders, guidelines and quality improvement measures based on evidence from randomized controlled clinical trials have been developed. Typically, these guidelines provide guidance for treatment of discrete chronic conditions. According to the 2008 World Health Organization (WHO) report [1], as many as 25% of 65–69 year olds and 50% of 80–84 year olds are affected by two or more chronic health conditions simultaneously. Recognizing the problem, and having estimated that in the USA over 25% of the general population has multiple chronic conditions (MCC), the National Quality Forum (NQF) under contract with the Department of Health and Human Services in the USA developed a measurement framework for individuals with MCC to be used in future NQF recommendations [2]. The epidemiology of MCC has been studied in several countries including Australia [3], Canada [4], Denmark [5], England [6], Sweden [7], Germany [8], the Netherlands [9], and most recently Scotland [10]. A common observation has been that the incidence of MCC increases as the population’s age increases and appears at an earlier age in deprived as opposed to wealthy populations. The implications of MCC are myriad, far reaching and differ depending on whether the perspective is that of society, the healthcare system, or the individual [2].

Care for the individual patient with MCC is often fragmented, split between providers leading to inefficient, incomplete and ineffective care. The individual with MCC is at risk for preventable adverse drug events [11], avoidable hospital admissions [12] and mortality [13]. Boyd et al. have estimated that a patient with five co-morbid conditions, treated according to clinical guidelines, would result in the prescription of 19 doses of 12 different medications taken at five time points during the day and carrying the risk of ten attendant interactions or adverse events [14]. Treatments for one disease can be considered either synergic or contradictory for other conditions. While trying to improve a patient’s health, one might actually be harming the patient in another aspect leading to increased hospitalization, increased burden on primary care physicians [5], increased consults and spiraling healthcare costs [15].

From a healthcare system perspective MCC is expensive [15] with a major part of the burden falling on the shoulders’ of primary care providers [6]. We have previously shown that in Israel hypertension, diabetes mellitus and female infertility treatments impose an economic burden comparable with that of cancer and cardiovascular diseases [16]. In the USA among Medicare insurers 80% of expenses are devoted to patients with four or more chronic conditions and costs increase exponentially as patients accrue chronic conditions [15]. MCC is thus a threat not only to the patient’s health but to healthcare systems’ economic sustainability as well.

Facing the complexity of MCC, and in order to plan an effective local strategy to deal with the issue of individual and organizational risk, the first step is to diagnose the extent and complexity of MCC at the local level. We therefore set out to examine MCC in the Maccabi Healthcare Service (MHS). Various measures have been used to define and measure MCC [2,17]. The National Quality Forum has recently proposed the following definition: People with two or more concurrent chronic conditions that collectively have an adverse effect on health status, function or quality of life and that require complex healthcare management, decision-making, or coordination [2]. For this study we chose to adopt the strategy recently presented by Barnett et al. [10] from Scotland, and looked at the prevalence of the same forty chronic conditions amongst our patients (Table 1). This comparison is relevant as Scotland is a nation of comparable size to Israel which also has a national health care insurance system. MHS, the second largest health management organization in Israel, provides care for two million enrollees (one quarter of the Israeli population), and thus is especially suited for such a study.

**Table 1:** Chronic conditions included in multiple chronic condition analysis

1	Hypertension	21	Atrial fibrillation
2	Depression	22	Peripheral vascular disease
3	Painful condition	23	Heart failure
4	Asthma	24	Prostate disorders
5	Coronary heart disease	25	Glaucoma
6	Treated dyspepsia	26	Epilepsy (currently treated)
7	Diabetes mellitus	27	Dementia
8	Thyroid disorders	28	Schizophrenia/psychosis and bipolar disorder
9	Rheumatoid arthritis, other inflammatory polyarthropathies & systematic connective tissue disorders	29	Psoriasis or eczema
10	Deafness/Hearing Loss	30	Inflammatory bowel disease
11	Chronic obstructive pulmonary disease	31	Migraine
12	Anxiety and other neurotic an stress related disorders	32	Blindness & low vision
13	Irritable bowel syndrome	33	Chronic sinusitis
14	Neoplasia/Cancer	34	Learning disability/ADHD
15	Alcohol problems	35	Anorexia or bulimia
16	Other psychoactive substance misuse	36	Bronchiectasis
17	Treated constipation	37	Parkinson’s disease
18	Stroke & transient ischemic attack	38	Multiple sclerosis
19	Chronic kidney disease	39	Chronic viral hepatitis
20	Diverticular disease of intestine	40	Chronic liver disease

## Methods

### Study design and participants

This cross-sectional study was conducted in MHS, which insures approximately two million members. In Israel, universal healthcare services are provided by four health maintenance organizations that are obligated to insure every citizen who wishes to join them irrespective of age, sex, or medical history. Therefore every sector in the Israeli population is represented in MHS. MHS has used electronic medical records for over twenty years, and a unified patient chart now allows all providers to view patient information on a “need-to-know” basis. MHS’ central databases are automatically updated with every member transaction including all physician visits, prescriptions dispensed, laboratory tests, medical treatments, nursing care, physiotherapy treatment, hospital admissions, outpatient visits or any other medical service rendered to the insured individual through MHS, who is issued a unique identification number.

### Data collection

The data for this analysis were collected from all active MHS members alive on August 6, 2012. The dataset included age, sex, and socioeconomic status. Socioeconomic status (SES) was categorized into ten levels according to the poverty index of the member’s enumeration area as defined by the 1995 national census based on several parameters, including household income, educational qualifications, crowding, material conditions and car ownership [18].

To assess MCC, we followed the method presented by Barnett et al. [10] and selected 40 disorders (Table 1), which were defined by clinical coding and prescription data. In addition to the diagnosis of learning disability we also coded for Attention Deficit Disorder (ADHD). For several morbid conditions we used data from MHS’ automated patients’ registries including the cancer registry, the diabetes mellitus patient registry [19,20], the cardiovascular disease registry [21], the hypertension registry [22] and the mental health registry [23]. These registries are updated daily and automatically utilizing strict algorithms. The algorithms draw data from numerous sources including physicians’ diagnoses, prescription information, data acquired from hospital discharge codes and billing information from providers. We defined multimorbidity (multiple chronic conditions; MCC) as the presence of two or more of these morbidities in one patient. Similar to Barnett et al. [10] we defined each disorder as either a physical or mental health disorder.

### Statistical analyses

We calculated age-specific prevalence and frequency of MCC and compared them to the results published by Barnett et al. [10] for the Scottish population. Analyses were stratified by socio-economic status ranging from 1 (highest) to 10 (lowest). Difference in age and sex distribution between MHS and the Scottish population were calculated by Chi-square test. Confidence intervals (CI) were calculated with Fisher’s exact method. Statistical analyses were conducted using IBM-SPSS statistics (version 20). The study protocol has been approved by the MHS Research Ethics Committee.

## Results

The total study population included in the present analysis was 1,972,798 individuals. Children and adults younger than 25 years accounted for 43% of this population compared to 27% in the Scottish study (Table 2) [10]. The Scottish study population has a higher proportion of elderly individuals (17%) compared to 8% in MHS (P < 0.01).

**Table 2:** **Characteristics of Maccabi Healthcare Services study population (year 2012) nd people registered with 314 medical practices in Scotland (2007) **[1]

		**MHS (n = 1972798)**		**Scotland (n = 1751841)**	
		**n**	**%**	**n**	**%**
Sex	F	1009745	51.2%	884420	50.5%
	M	963053	48.8%	867421	49.5%
Age	<=24	842069	42.7%	479156	27.4%
	25-44	574386	29.1%	508389	29.0%
	45-64	405067	20.5%	473127	27.0%
	65-84	135679	6.9%	254600	14.5%
	85+	13468	0.7%	36569	2.1%
MCC	0	686406	34.8%	1012980	57.8%
	1	534222	27.1%	333365	19.0%
	2	311986	15.8%	167518	9.6%
	3	173749	8.8%	99487	5.7%
	4	101292	5.1%	60417	3.4%
	5	62161	3.2%	35641	2.0%
	6	39962	2.0%	20507	1.2%
	7	25284	1.3%	11080	0.6%
	8 or above	37736	1.9%	10846	0.6%

MCC, multimorbidity.

**Figure 1:**
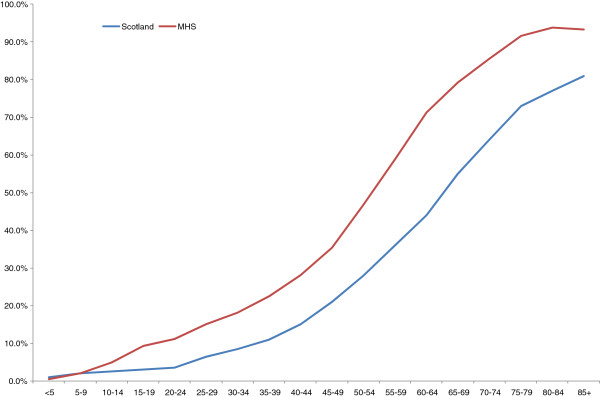
**Age-specific prevalence of multimorbidity in MHS and Scotland **[10]**.**

A total of 65.2% (95% CI 65.1%-65.3%) of the population had one or more chronic morbidities, and 38.1% (38.0%–38.2%) had two or more chronic disorders, i.e. MCC. These are significantly (P < 0.01) higher rates compared with 42.2% (42.1%-42.3%) and 23.2% (23.1%-23.3%) in the Scottish study population, respectively. In the age-specific analysis, the prevalence of MCC increased with age from 0.4% in infants and young children aged under five years to 28.1% in the 40-44 year age group, to over 90% after age 75 (Figure 1). While similar results were calculated for children, the respective prevalence rates among the adult Scottish study population were 15% for 40-44 year olds and 81% after age 85.

**Figure 2:**
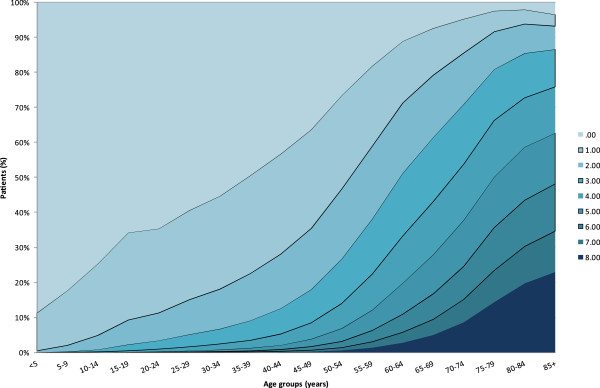
Number of chronic disorders by age-group.

The number of disorders per patient increased substantially with increasing age (Figure 2). In the 45-49 year age group, two thirds (63.7%) of the population had at least one chronic disorder, and one third (35.5%) had two or more morbidities (MCC). Between ages 70-74, 95.2% of the group had at least one chronic disorder and 85.5% had MCC. One quarter of the younger adolescents (10-14 years old) and one third of the older adolescents (15-19 years old), had at least one chronic condition. The most prevalent disorder in these age groups was learning disabilities found in 8.7% of the younger adolescents (10-14 year olds) and 6.2% of the older adolescents (15-19 year olds), respectively. Asthma and other pulmonary diseases were reported in 0.7% and 6.1% of these age groups, respectively.

**Figure 3:**
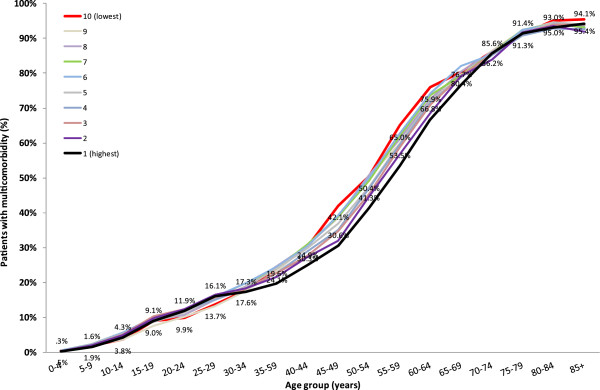
Prevalence of multimorbidity by age and socioeconomic status.

Residing in high SES level was associated with a low prevalence of MCC, particularly between age 35 and 65 years (Figure 3). In the 45- 49 age group, MCC was recorded in 42.1% of the individuals in the lowest SES compared with 30.6% in the highest level. The respective prevalence rates in the Scottish population were 26.8% and 13.4% for this age group. No substantial differences were observed in the older age groups, while among young adolescents (10-14 years old) a positive association was calculated between SES level and MCC prevalence (3.8% in the lowest SES level compared to 4.3% in the highest level).

## Discussion

In our large cohort, representing approximately one quarter of the Israeli population, MCC is very prevalent, increases with age and with the exception of children and adolescents is more prevalent in lower than higher socioeconomic strata. Not only is MCC associated with age, the severity of MCC, as determined by the number of chronic disorders accrued per person, increases with age. Of special interest are the young age at which MCC becomes evident, and its distribution in our cohort. The main diagnoses contributing to MCC in children in our cohort were asthma, learning disabilities and visual disturbances. The high prevalence of asthma in young children in our population, is in accordance with data from the USA where a diagnosis of asthma was given to 8.3%, 14.4% and 18.7% of children in age groups <5, 5-11 and 12-17 year old, respectively [24]. Additionally in our population asthma was more prevalent in lower SES groups [24]. In addition to a high prevalence of asthma (6.8%), children aged 5-10 in our population were diagnosed as having learning disabilities (5.8%) and visual impairment (2.1%) leading to MCC at a very young age.

Of particular interest in our cohort is the distribution of MCC across socioeconomic status and patient age. In contrast with previously reported studies, higher prevalence of MCC was recorded in higher socioeconomic status groups, particularly amongst adolescents and young adults. This difference was mostly driven by the diagnosis of learning disabilities. One explanation could be that having a recorded diagnosis of learning disability entitles student patients to adjustments and extra time in high school and university examinations [25]. The diagnosis must be provided after didactic, psychological and/or medical diagnosis, a process that is more easily accessed by those with means through the private sector, leading to a bias of the wealthy in this regard. The higher prevalence of learning disorders in younger than older children might be explained by an increase emphasis put on diagnosing this disorder in Israeli society in recent years. After the age of 30 the difference in MCC amongst the SES groups per age is in the order of 10% or less (a much tighter range than that observed in the Scottish population) attesting to a socially equitable health system in Israel.

Compared to the data from Scotland [10] we found a higher prevalence of MCC across all age groups. Is our population truly more ill than the Scottish population, have the MHS cohort been over-diagnosed, or have diagnoses been made in a more conservative mode in the Scottish cohort? One possibility could be that MHS has been using electronic databases for over two decades, thus diagnoses of chronic disease are not lost. The answer though is not possible to say from the data at hand, an additional study designed to answer the difference would be needed, yet the comparison of the two populations does lead to some interesting observations.

At ages 50-54 the rate of MCC was double that which was found in the Scottish cohort (46.7% vs 28%). Although in this paper we took the same approach in evaluating the data as Barnett et al. [10], differences in the way diagnoses are recorded in the two systems may account for some of the differences between the results of the two studies. In our system every medical condition is recorded in a unified electronic medical record that tracks chronic medical conditions throughout the lifespan of citizens. This recording method that has been used during the last twenty years lends itself to accumulation of chronic conditions from young age.

In comparison with the Scottish population described by Barnett et al. [10] our study population is much younger. The Israeli population in general is younger than the average age of the Organization for Economic Co-operation and Development (OECD) nations. At the end of 2011 28.2% of the Israeli population was younger than 15 years old in comparison with the average of the OECD nations of 18.5%. Additionally in Israel only 10.3% and less were older than 65 years compared with a 15% average for the OECD nations [26]. Although the data are age specific, differences in the rate of diagnoses of chronic diseases may be attributed to differences in care practice for children in Israel. Despite the fact that both the Israeli and the British healthcare care systems provide universal health insurance with a very generous health basket and low copayments for primary and secondary care, there are some differences. According to the 2013 OECD report, the percentage of pediatricians amongst Israeli physicians is almost double that in the United Kingdom (9.38% in Israel versus 5.43% in the UK) and the density of pediatricians per 1,000 population is also double (0.3 in Israel versus 0.15 in the UK) [27]. The high prevalence of learning disabilities in Israel, perhaps, is enabled by high accessibility to pediatric care combined with a tendency of wealthier parents to establish the condition given the advantages in school that it entitles to children.

The prevalence of MCC presents challenges to modern health systems and providers. In this regard, the use of integrated electronic medical records made it possible for provider and health care system managers to comprehend the magnitude and complexity of MCC. The challenges to providers are significant, for example in trying to use the fewest necessary drugs possible, with the least side effects, to devise a personalized effective and safe treatment plan for each patient. Healthcare system managers are interested in optimizing the use of scarce resources efficiently in order to obtain maximal health of the population. In this regard the study of local patterns of MCC is of utmost importance.

In order for healthcare systems to effectively approach the treatment of chronic disorders, the multidimensional Chronic Care Model (CCM) was developed by Wagner et al. [28]. Though this model was subsequently thought to be both efficacious and cost-reducing [29], the phenomenon of MCC, not covered in the original CCM or by most other treatment guidelines, has emerged as one of the most important challenges to modern healthcare systems.

Management must provide caretakers with new, more complex educational tools and supportive information technology to help achieve best possible results. This should include seminars and lectures not only on discrete diseases but also on the efficient management of disease combinations such as diabetes mellitus, hypertension, hyperlipidemia and chronic renal failure. A recent Cochrane review which looked at studies examining interventions for improving outcomes in patients with MCC in primary care and community settings, concluded that though the research in the field is scarce, there are indications that interventions targeted at either specific combinations of diseases, or at specific problems for patients with multiple conditions may be most effective [30].

General practitioners find consultations with multi-morbid patients demanding and not easily delegated to nurses [5], attesting to the high work burden currently faced by caregivers, a burden which can be expected to increase in coming years. It can be expected that along with an increased workload, physicians will experience increased burnout, and it is of utmost importance that health management systems set up support systems for their caretakers in order to minimize this phenomenon. New systems of healthcare need to be devised to handle the needs of patients with MCC. New decision support tools for caregivers based on information technologies are needed at point of care. Undoubtedly future healthcare systems dealing with MCC will include team work that will encompass a wide variety of medical and paramedical caregivers including, but not limited to, physicians and nurses along with dieticians, social workers, physiotherapists, clinical pharmacologists and consultant physicians all working in unison to adjust goals and comprehensive treatment plans to meet the goals and demands of the individual patient with MCC. Moreover it is expected that new technologies utilizing telemedicine and advanced technologies will be implemented in the care of these patients. Tools that help patients cope with the complex medical instructions and that enable better active participation in the monitoring and managing of MCC are needed.

A limitation to our study is the use of census data from 1995 in analysis of the association of MCC with socioeconomic status. It will be important to update our analysis as new and reliable census data becomes available.

## Conclusions

The current data indicates that MCC is highly prevalent in all age groups in Israel. A high prevalence of MCC has far-reaching implications in the planning of health care systems. As patients live longer lives and accrue chronic disorders, both mental and physical, the management of combinations of these disorders becomes more and more complex. There is therefore an increasing need for tertiary as well as secondary and primary prevention to prevent poor outcomes of combinations of chronic disorders. Finally, but most important, as succinctly pointed out to by Guthrie et al. [31] clinical guidelines which provide the basis of modern day clinical care must be improved and adapted to account for patients with MCC.

## Competing interests

The authors declare that they have no competing interests.

## Authors’ contribution

JEA participated in the design of the study, interpretation of the results, drafted the manuscript and made critical revisions; GC participated in the design of the study, performed the statistical analysis, and helped to draft the manuscript; AG carried out the data extraction, participated in the statistical analysis, drafting of the manuscript and made critical revisions; AP conceived the study, participated in its design helped to draft the manuscript and critical revisions. All authors read and approved the final manuscript.

## Authors’ information

JEA is Deputy Medical Director of the Southern Region of Maccabi Healthcare Services and Lecturer in Medicine at the Ben Gurion University of the Negev; GC is Head of the Epidemiology & Database Research Unit at Maccabi Healthcare Services, and Associate Professor at the Tel-Aviv University Faculty of Medicine AG, Data analyst in the Department of Medical Informatics Maccabi Healthcare Services; AP is Director of Maccabi Institute for Health Services Research and professor of medicine at the Ben Gurion University of the Negev.

## References

[B1] The World Health Report 2008 - Primary Health Care (Now More Than Ever) 2008

[B2] National Quality Forum Multiple Chronic Conditions Measurement Framework National Quality Forum: Multiple Chronic Conditions Measurement Framework 2012 Washington DC: National Quality Forum

[B3] Taylor AW Price K Gill TK Adams R Pilkington R Carrangis N Shi Z Wilson D Multimorbidity - not just an older person’s issue. Results from an Australian biomedical study BMC Public Health 2010 10 718 10.1186/1471-2458-10-718 21092218 PMC3001730

[B4] Agborsangaya CB Lau D Lahtinen M Cooke T Johnson JA Multimorbidity prevalence and patterns across socioeconomic determinants: a cross-sectional survey BMC Public Health 2012 12 201 10.1186/1471-2458-12-201 22429338 PMC3353224

[B5] Moth G Vestergaard M Vedsted P Chronic care management in Danish general practice–a cross-sectional study of workload and multimorbidity BMC Fam Pract 2012 13 52 10.1186/1471-2296-13-52 22676446 PMC3436724

[B6] Salisbury C Johnson L Purdy S Valderas JM Montgomery AA Epidemiology and impact of multimorbidity in primary care: a retrospective cohort study British J Gen Pract: J Royal College of Gen Pract 2011 61 e12 e21 10.3399/bjgp11X548929 PMC3020068 21401985

[B7] Marengoni A Winblad B Karp A Fratiglioni L Prevalence of chronic diseases and multimorbidity among the elderly population in Sweden Am J Public Health 2008 98 1198 1200 10.2105/AJPH.2007.121137 18511722 PMC2424077

[B8] van den Bussche H Koller D Kolonko T Hansen H Wegscheider K Glaeske G von Leitner EC Schafer I Schon G Which chronic diseases and disease combinations are specific to multimorbidity in the elderly? Results of a claims data based cross-sectional study in Germany BMC Public Health 2011 11 101 10.1186/1471-2458-11-101 21320345 PMC3050745

[B9] van Oostrom SH Picavet HS van Gelder BM Lemmens LC Hoeymans N van Dijk CE Verheij RA Schellevis FG Baan CA Multimorbidity and comorbidity in the Dutch population - data from general practices BMC Public Health 2012 12 715 10.1186/1471-2458-12-715 22935268 PMC3490727

[B10] Barnett K Mercer SW Norbury M Watt G Wyke S Guthrie B Epidemiology of multimorbidity and implications for health care, research, and medical education: a cross-sectional study Lancet 2012 380 37 43 10.1016/S0140-6736(12)60240-2 22579043

[B11] Field TS Gurwitz JH Harrold LR Rothschild J DeBellis KR Seger AC Auger JC Garber LA Cadoret C Fish LS Garber LD Kelleher M Bates DW Risk factors for adverse drug events among older adults in the ambulatory setting J Am Geriatr Soc 2004 52 1349 1354 10.1111/j.1532-5415.2004.52367.x 15271125

[B12] Condelius A Edberg AK Jakobsson U Hallberg IR Hospital admissions among people 65+ related to multimorbidity, municipal and outpatient care Arch Gerontol Geriatr 2008 46 41 55 10.1016/j.archger.2007.02.005 17403548

[B13] Gijsen R Hoeymans N Schellevis FG Ruwaard D Satariano WA van den Bos GA Causes and consequences of comorbidity: a review J Clin Epidemiol 2001 54 661 674 10.1016/S0895-4356(00)00363-2 11438406

[B14] Boyd CM Darer J Boult C Fried LP Boult L Wu AW Clinical practice guidelines and quality of care for older patients with multiple comorbid diseases: implications for pay for performance JAMA 2005 294 716 724 10.1001/jama.294.6.716 16091574

[B15] Wolff JL Starfield B Anderson G Prevalence, expenditures, and complications of multiple chronic conditions in the elderly Arch Intern Med 2002 162 2269 2276 10.1001/archinte.162.20.2269 12418941

[B16] Chodick G Porath A Alapi H Sella T Flash S Wood F Shalev V The direct medical cost of cardiovascular diseases, hypertension, diabetes, cancer, pregnancy and female infertility in a large HMO in Israel Health Policy 2010 95 271 276 10.1016/j.healthpol.2009.12.007 20061044

[B17] Huntley AL Johnson R Purdy S Valderas JM Salisbury C Measures of multimorbidity and morbidity burden for use in primary care and community settings: a systematic review and guide Ann Fam Med 2012 10 134 141 10.1370/afm.1363 22412005 PMC3315139

[B18] Census of Population and Housing[http://www.cbs.gov.il/mifkad/hesber/mavo13.doc]

[B19] Heymann AD Chodick G Halkin H Kokia E Shalev V [Description of a diabetes disease register extracted from a central database] Harefuah 2007 146 15 17 79 17294841

[B20] Chodick G Heymann AD Shalev V Kookia E The epidemiology of diabetes in a large Israeli HMO Eur J Epidemiol 2003 18 1143 1146 14758871 10.1023/b:ejep.0000006635.36802.c8

[B21] Shalev V Chodick G Goren I Silber H Kokia E Heymann AD The use of an automated patient registry to manage and monitor cardiovascular conditions and related outcomes in a large health organization Int J Cardiol 2011 152 345 349 10.1016/j.ijcard.2010.08.002 20826019

[B22] Chodick G Porath A Alapi H Sella T Flash S Wood F Shalev V The direct medical cost of cardiovascular diseases, hypertension, diabetes, cancer, pregnancy and female infertility in a large HMO in Israel Health policy (Amsterdam, Netherlands) 2010 95 271 276 10.1016/j.healthpol.2009.12.007 20061044

[B23] Kodesh A Goldshtein I Gelkopf M Goren I Chodick G Shalev V Epidemiology and comorbidity of severe mental illnesses in the community: findings from a computerized mental health registry in a large Israeli health organization Soc Psychiatry Psychiatr Epidemiol 2012 47 1775 1782 10.1007/s00127-012-0478-9 22310700

[B24] Bloom B, Cohen RA, Freeman G: Summary health statistics for U.S. children: National Health Interview Survey National Center for Health Statistics Vital Health Stat 2011 2012 10 25116332

[B25] Hozer Mancal Misrad Hahinuch Bagrut examination and learning disabilities Book Hozer Mancal Misrad Hahinuch, Bagrut examination and learning disabilities 2003 http://cms.education.gov.il/EducationCMS/applications/mankal/arc/sd4bk4_3_25.htm

[B26] Israeli Central Bureau of Statistics News Release Demographic Situation in Israel Book Israeli Central Bureau of Statistics News Release: Demographic Situation in Israel 2011 http://www.cbs.gov.il/reader/newhodaot/hodaa_template.html?hodaa=201301008

[B27] OECD Health Data 2013, online database http://www.oecd.org/health/health-systems/oecdhealthdata.htm (accessed June 27 2013)

[B28] Wagner EH Austin BT Davis C Hindmarsh M Schaefer J Bonomi A Improving chronic illness care: translating evidence into action Health Aff (Millwood) 2001 20 64 78 10.1377/hlthaff.20.6.64 11816692

[B29] Bodenheimer T Wagner EH Grumbach K Improving primary care for patients with chronic illness: the chronic care model, Part 2 JAMA 2002 288 1909 1914 10.1001/jama.288.15.1909 12377092

[B30] Smith SM Soubhi H Fortin M Hudon C O’Dowd T Interventions for improving outcomes in patients with multimorbidity in primary care and community settings Cochrane Database Syst Rev 2012 4 CD006560 10.1002/14651858.CD006560.pub2 22513941

[B31] Guthrie B Payne K Alderson P McMurdo ME Mercer SW Adapting clinical guidelines to take account of multimorbidity BMJ 2012 345 e6341 10.1136/bmj.e6341 23036829

